# Coverage, Diversity, and Functionality of a High-Latitude Coral Community (Tatsukushi, Shikoku Island, Japan)

**DOI:** 10.1371/journal.pone.0054330

**Published:** 2013-01-14

**Authors:** Vianney Denis, Takuma Mezaki, Kouki Tanaka, Chao-Yang Kuo, Stéphane De Palmas, Shashank Keshavmurthy, Chaolun Allen Chen

**Affiliations:** 1 Biodiversity Research Center, Academia Sinica, Nangang, Taipei, Taiwan; 2 Kuroshio Biological Research Foundation, Nishidomari, Otsuki-cho, Kochi, Japan; 3 Taiwan International Graduate Program-Biodiversity, Academia Sinica, Nangang, Taipei, Taiwan; 4 Institute of Oceanography, National Taiwan University, Taipei, Taiwan; Leibniz Center for Tropical Marine Ecology, Germany

## Abstract

**Background:**

Seawater temperature is the main factor restricting shallow-water zooxanthellate coral reefs to low latitudes. As temperatures increase, coral species and perhaps reefs may move into higher-latitude waters, increasing the chances of coral reef ecosystems surviving despite global warming. However, there is a growing need to understand the structure of these high-latitude coral communities in order to analyze their future dynamics and to detect any potential changes.

**Methodology/Principal Findings:**

The high-latitude (32.75°N) community surveyed was located at Tatsukushi, Shikoku Island, Japan. Coral cover was 60±2% and was composed of 73 scleractinian species partitioned into 7 functional groups. Although only 6% of species belonged to the ‘plate-like’ functional group, it was the major contributor to species coverage. This was explained by the dominance of plate-like species such as *Acropora hyacinthus* and *A. solitaryensis*. Comparison with historical data suggests a relatively recent colonization/development of *A. hyacinthus* in this region and a potential increase in coral diversity over the last century. Low coverage of macroalgae (2% of the benthic cover) contrasted with the low abundance of herbivorous fishes, but may be reasonably explained by the high density of sea urchins (12.9±3.3 individuals m^−2^).

**Conclusions/Significance:**

The structure and composition of this benthic community are relatively remarkable for a site where winter temperature can durably fall below the accepted limit for coral reef development. Despite limited functionalities and functional redundancy, the current benthic structure might provide a base upon which a reef could eventually develop, as characterized by opportunistic and pioneer frame-building species. In addition to increasing seawater temperatures, on-going management actions and sea urchin density might also explain the observed state of this community. A focus on such ‘marginal’ communities should be a priority, as they can provide important insights into how tropical corals might cope with environmental changes.

## Introduction

As chances to limit global temperature increase to <2°C by the end of the century seem seriously compromised [1], tropical reef corals (hereafter ‘corals’) must demonstrate exceptional acclimatization and/or adaptation capacities in order to survive future environmental changes [2]. Most species may fail to develop mechanisms to survive future conditions, and worldwide declines in coral reefs now seem inevitable [3]. Poleward shifts of coral reef development at current latitudinal limits may be a reasonable scenario for coral reef survival [4], and thus subtropical areas may be viewed as potential refuge habitats [5].

Corals can tolerate water temperatures of as low as 14°C for a short period of time, but coral reef development is usually considered to be limited by temperatures of <18°C [6]. Reduced growth and reproductive abilities [7] and increased competition with macroalgae [8] are likely to reduce the development of coral communities at high latitudes. Coral communities thus usually fail to form limestone reef structures at high latitudes [9] and are considered ‘marginal’ [10]. With climate change, rapid and fundamental changes of temperate coastal ecosystems may have already begun at temperate coastal ecosystems [11]–[13] accompanied by changes in distributions of tropical coral reefs. However, data are still lacking on potential rates of such expansion under rapid environmental change [2], and it is still unclear if these reefs can function as suitable and sustainable habitats for tropical species [5].

Japan extends from tropical (20.42°N) to temperate (45.55°N) latitudes. The Kuroshio Current (KC), the world’s strongest continental boundary current [14], strongly influences the ecology of this bioregion as it flows along the Japanese coastline that faces the Pacific Ocean. This warm 100-km-wide current extends from the Philippines to the Japanese archipelago and moves at an average speed of 6∼7 km h^−1^ [15]. By maintaining the average temperature above the minimum temperature required for coral reef survival, it allows corals to colonize more-northerly locations compared to other regions at similar latitudes [6]. Coral assemblages in Japan were divided by Veron [16] into 3 groups: tropical reefs (southern Islands of the Ryukyus Archipelago), temperate non-reefal communities (most of the coast along the main islands of Japan), and outlying communities (northernmost limit of coral distribution around Tokyo). Fossil records from the latter region show that during the mid-Holocene (i.e., 6000∼5000 years ago) when seawater temperature were <2°C higher than at present, reefs were much more prolific along the coast of Japan [17]. This suggests that environmental conditions (i.e., salinity, nutrients, light availability, aragonite saturation, waves, currents, and storm frequency [18]) of these areas were and could again become particularly favorable for reef development as seawater temperatures increase.

Shikoku (33.75°N) is the smallest of Japan’s four main islands. Corals are more abundant on the west than east coast of this island, and the area of Tatsukushi is known to possess the highest abundance of corals in the region [19]. In 1970, Tatsukushi was the first area in Japan to be designated a marine park zone [20]. In the past, coral communities from this area demonstrated particularly high resilience capacities to sedimentation [21]. Areas such as this which are below the usual thermal limit for coral reef development (i.e., the 18°C isotherm [18]) are important areas to document, particularly in the context of future ecosystem shifts due to predicted environmental changes [22]–[24].

The main goal of the present study was to analyze the structure of the benthic community located at the high-latitude Tatsukushi site. We examined the benthic composition and diversity of major organisms present at this site to document the current state of this ‘marginal’ community. We analyzed ecological functions presented by this community according to the role of different species in the ecosystem, and explained the observed state in light of recent studies suggesting the poleward expansion of tropical reef corals in Japan in response to rising sea surface temperatures.

## Materials and Methods

### Ethics Statement

This was a collaborative work between the Biodiversity Research Center, Academia Sinica, Taiwan and the Biological Institute of Kuroshio (BIK), Japan. The BIK was in charge of routine surveys for the Marine Park of Tatsukushi, and since we were working in collaboration with the BIK and collected no coral samples for this research, there was no need to obtain formal permission from the Marine Park authorities. All survey procedures carried out were non-destructive and were done with proper precautions to prevent any damage to the ecosystem.

### Study Site

**Figure 1:**
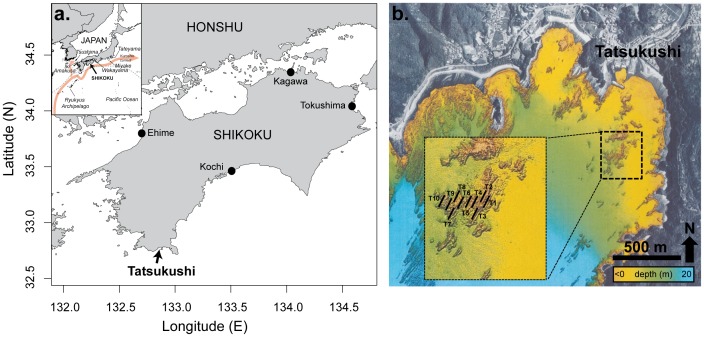
Study site. a. Location of Tatsukushi at Shikoku Island (Japan). **b.** Local geographic features of Tatsukushi Bay with the area survey and approximate locations of transects. Color scale corresponds to bathymetry obtained by sonar exploration [25].

At Tatsukushi (Shikoku, southern Japan, Figure 1a), a well-developed high-latitude (32.75°N) coral community is located on patchy rocks at a distance of ∼250 m from the shore and ∼600 m from the entrance to Tatsukushi Harbor (Figure 1b). The maximum length and width of this area is ∼220 m, and total surface cover is approximately 2.1 ha. Upper parts of these patchy rocks reach 3 m in depth, but most of the structure is found at around 5 m and slopes down to 8∼10 m in depth. It is surrounded by sandy and muddy areas where rocks emerge from the sediments, constituting a hard substrate available for settlement of sessile organisms. Tatsukushi Bay was the subject of a restoration project since 2002 as a consequence of severe damage caused to the coral reef communities from high sedimentation following heavy rains which occurred in 2001.

Ninety-six scleractinian species are present in the general area of Tatsukushi [21]. In 2010, a visual survey of the algal biodiversity at the study site recorded 17 species of macroalgae [25]. The fish community, surveyed on 10 sections of 40 m^2^ along a 100-m transect (belt transect of 10×4 m with 4 observers), was composed of 91 species [25]. Trophic levels of fishes and their functions in the ecosystem was used to classify each species into functional categories according to the information available on FishBase [26].

### Seawater Temperatures

**Figure 2:**
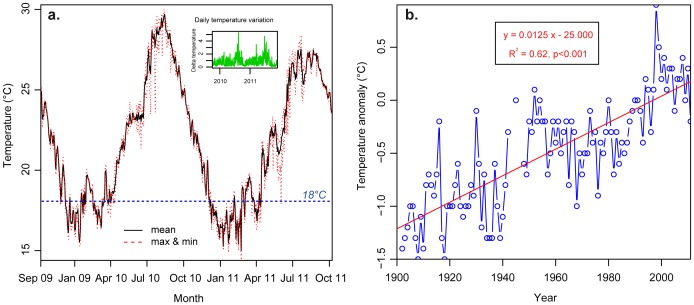
Seawater temperatures. a. Mean, maximum, and minimum seawater temperatures at the study site (at 5 m in depth) from November 2009 to October 2011. Inset represents daily temperature variations for the same period (data from [21], [25]). **b.** Long-term trend in sea surface temperature anomalies (deviation of mean annual temperature from mean temperature of the preceding three decades) from 1902 to 2011 in the northern part of the seas south of Japan. Red line corresponds to the linear regression analysis showing the +1.25°C increase occurring over the last century (data from [37]).

The KC strongly influences seawater temperatures of the region. Seawater temperature (to an accuracy of 0.2°C) was recorded at 5 m in depth from November 2009 to October 2011 at hourly intervals using calibrated underwater temperature loggers (Hobo Water Temp Pro, Onset Computer Corp., Pocasset, USA, Figure 2a). The mean temperature was 22.4±3.9°C with a monthly average ranging from 28.4°C (September 2010) to 16.8°C (February 2011). The maximum temperature was recorded in September 2010 (30.0°C) and the minimum temperature in March 2011 (14.4°C). Daily temperature variations (delta temperature) are low (0.8±0.6°C), except during the typhoon season when it reached 5.3°C (inset in Fig. 2). During the winters of 2009/2010 and 2010/2011, 70 and 94 days respectively presented a daily average temperature under the accepted limit for coral reef development (<18°C).Historical data of sea surface temperatures (SSTs) from 1902 to 2011 were provided by the Japan Meteorological Agency (www.jma.go.jp/jma/indexe.html). SSTs data from the northern part of the seas south of Japan (bordering Shikoku) are obtained from various kinds of oceanographic (research vessels, profiling floats, buoys) and satellite observations, as well as voluntary reports from ships. Temperature anomalies were calculated by comparing annual mean of SSTs (n = 110) to the average means of the 3 last decades. Linear regression analysis over the last century shows an increase of +1.25°C in the SSTs in the seas bordering Shikoku Island to the south (Figure 2b).

### Benthic Survey

In September 2011, ten 20-m line transects (Figure 1b) were used to assess the benthic community by analyzing photographs (0.5×0.5 m) taken regularly at 1-m intervals (21 photographs per transect). The total surface area examined was 52.5 m^2^. Transects were placed haphazardly at a ∼5-m depth, with a minimum distance of 5 m separating any 2 transects. Fifty random points were positioned on each picture, and the underlying benthic category was identified. Seven major categories (corals, algae, coralline algae, other live organisms, limestone, rock, and unstable substrate) were used to describe the benthic cover. Sea urchin density was estimated by recording the total number of sea urchins in each photograph. Coral diversity (sensu [6]) was then assessed by visual inspection by a coral taxonomist (T. Mezaki) during the same period and by repeated dives at the study site until no further species were identified (∼ 4 h).

Corals create habitats for many organisms and contribute to reef growth as framework builders [27]. Functional groups can be classified according to the shape of the colonies, reflecting their roles in these reef processes [28]. Each coral species was thus assigned to one or more of the 8 functional groups: massive, encrusting, foliose, columnar, plate-like, bushy, arborescent, and unattached, defined by a colony’s growth forms [6], [28]–[30].

## Results

**Figure 3:**
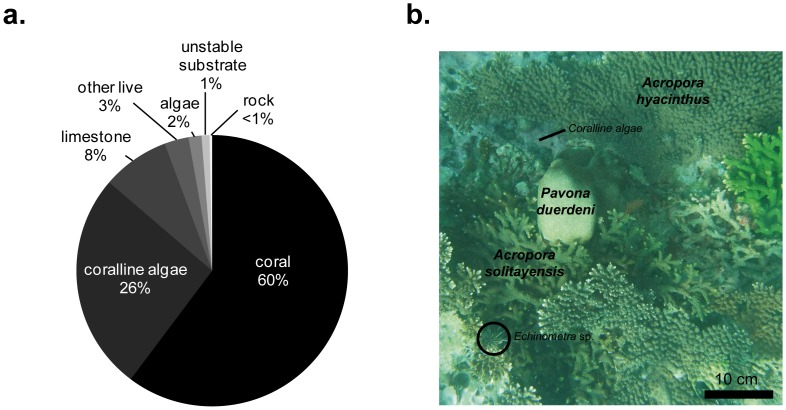
Benthic community. a. Percentage cover of major benthic categories. **b.** Appearance of coral community at Tatsukushi dominated by *Acropora* spp (at 5 m in depth).

The mean coral cover at this high-latitude site was 60±2% (Figure 3a) and even reached 74% on one of the transects. Other major categories that significantly contributed to the benthic cover were coralline algae (26%) and limestone (composed of dead coral, 8%). The ‘other live organism’ category (constituted mainly by sponges) accounted for 3% of the benthic cover, algae (turf- and macro-algae combined) for 2%, and bare substrate (rock or unstable) only <2% of the cover (Figure 3a).

Seventy-three species of scleractinian corals have been identified at the study site (Table S1). The Faviidae was the most diversified scleractinian family represented by more than 35% of species. However, in terms of benthic coverage, *Acropora* spp. were the dominant taxa (Figure 3b) with 2 species, *A. hyacinthus* and *A. solitaryensis,* being particularly abundant (together comprising 48.36% of the benthic community). Together, they contributed to >80% of the coral cover (Table 1).

**Table 1:** Contributions of different coral categories.

Species	Mean cover (%)	Standard error (%)
*Acropora hyacinthus*	35.78	4.46
*Acropora solitaryensis*	12.58	3.77
*Acropora* sp1	2.06	0.50
*Acropora* sp2	1.85	1.08
*Sinularia* sp*	1.79	0.56
*Stylocoeniella guentheri*	0.59	0.23
*Acropora valida*	0.27	0.19
*Acropora japonica*	0.22	0.18
*Hydnophora exesa*	0.21	0.18
*Acropora pruinosa*	0.19	0.14
*Pavona decussata*	0.19	0.10
*Porites lutea*	0.18	0.15
*Acropora* sp	0.17	0.11
*Acanthastrea echinata*	0.15	0.08
*Favia speciosa*	0.14	0.08
*Cyphastrea serailia*	0.13	0.08
*Favites flexuosa*	0.11	0.06
*Symphyllia radians*	0.09	0.09
*Echinophyllia aspera*	0.09	0.06
*Pocillopora damicornis*	0.09	0.04
*Turbinaria peltata*	0.09	0.09
*Pavona varians*	0.08	0.07
*Leptastrea pruinosa*	0.07	0.05
*Acropora willisae*	0.07	0.05
*Lobophytum* sp*	0.07	0.04
*Acropora nana*	0.06	0.06
*Stylophora pistillata*	0.06	0.06
*Montastrea valenciennesi*	0.05	0.03
*Pavona cactus*	0.04	0.04
*Coscinaraea crassa*	0.04	0.03
*Favites pentagona*	0.04	0.02
*Euphyllia ancora*	0.03	0.03
*Pavona duerdeni*	0.03	0.03
*Goniastrea favulus*	0.02	0.01
*Pectinia ayleni*	0.01	0.01
*Platygyra contorta*	0.01	0.01
*Cyphastrea* spp	0.97	0.36
*Acropora* spp	0.23	0.09
*Favites* spp	0.14	0.08
*Favia* spp	0.07	0.06
*Pavona* spp	0.05	0.05
*Turbinaria* spp	0.04	0.02
*Goniastrea* spp	0.01	0.01
Hard coral spp	0.81	0.24
Soft coral spp*	0.01	0.01
Recently dead coral	0.31	0.17

Based on an analysis of 10,500 points –50 random points by photographs).

* Soft coral species.

**Figure 4:**
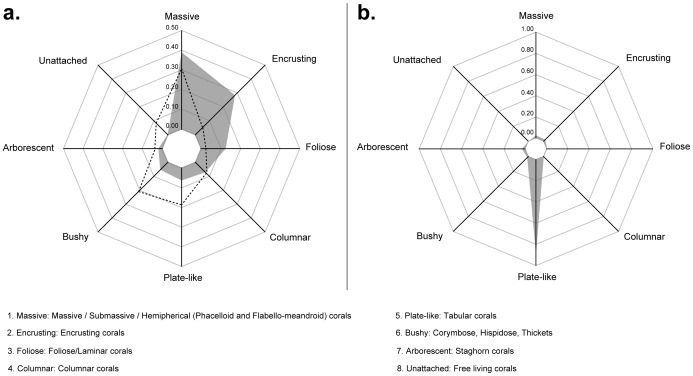
Functional composition of the coral assemblage at the study site. Based on **a.** the occurrence of species (the dotted line represents the functional composition of the Great Barrier Reef corals assemblage, from [28]) and **b.** the abundance of species. Axes represent the relative contribution of each of the 8 functional groups identified according to their roles in ecosystem processes.

Of the 73 species identified, 39% were massive, 28% encrusting, 12% foliose, 7% columnar, 6% plate-like, 5% bushy, and 2% arborescent (Figure 4a). Free-living or solitary corals (the ‘unattached’ functional group) were the only functional group not recorded. However, although only 6% of species belonged to the ‘plate-like’ functional group, this group was the major contributor to species coverage (Figure 4b). This can be explained by the over-dominance of plate-like species such as *A. hyacinthus* and *A. solitaryensis*.

At Tatsukushi, the fish community was largely dominated by carnivorous species: 73.6% of species were mainly carnivores, 19.8% herbivores, and 6.6% omnivores. Carnivorous fishes represented >93.7% of the total individuals recorded (*n* = 3142). Based on the analysis of the photographs taken for the benthic survey, the mean density of sea urchins was 12.9±3.3 individuals (ind) m^−2^.

## Discussion

Coral cover (60±2%) at this site largely exceeds common values recorded for reefs across the Indo-Pacific. On 390 reefs surveyed in 2003 across this region, coral cover averaged only 22.1% and only 7 reefs had a coral cover of >60% [31]. Only a few data have been published and are available to the international scientific community on the structure and composition of coral reef communities in Japan, particularly along its 4 main islands. Coral coverage of 18.2% was reported from Miyake-jima, near Tokyo at 34.05°N in 1979 [32], and Nozawa et al. [33] recorded coral coverage of 28.3% at Amakusa at 32.1°N in 2001. While most coral reef scientists know of examples of reefs with high coral cover (>60%) around the world, the latitude of Tatsukushi and its location beyond the accepted limit for coral reef formation [6], [16], [18] make the value reported here particularly exceptional. Moreover, the dominance of low-latitude species considered to be more thermally sensitive [9], [13], [34] distinctively differed from common descriptions of high-latitudinal reefs dominated by Faviid species [13], [32]–[35].

The KC is responsible for the development of non-reefal communities at these high latitudes and outlying populations further north in Japan, reaching as far as Tateyama, near Tokyo [9]. These waters where winter temperatures can fall below 18°C are generally considered to be the limit for tropical coral reef formation [6], [16], [18]. Based on these theoretical considerations of temperature, the latitudinal limit for coral reef development is usually reported to be the northern part of the Ryukyus Archipelago [36], although coral reefs are reported at up to 34°N at Tsushima Island, Japan [34]. The +1.25°C increase in seawater temperature observed over the last 100 years in southern parts of Shikoku Island (see [37] and Figure 2b) and the shortened duration of low temperatures suggest a possibility for poleward migration of coral reefs and associated organisms [13], [38]. Moreover, calcification, which is sometimes considered a limiting factor for coral reef development at high latitudes [39], [40] now seems to benefit these marginal reefs, which are experiencing warmer conditions [41]. Thus, connectivity along the KC may play a critical role in the expansion of coral reef distributions toward high-latitude areas (reviewed in [42]).

The rise above the seafloor (based on the vertical accretion of corals) of the coral community of this study was lacking, and it is debatable whether this community should be considered as a reef (see [43]). In spite of this, the current benthic state (60% corals, 26% coralline algae, and 8% limestone) and the low proportion (<2%) of bare substrate such as rocks and rubble suggest the potential frame-building role of this community. Fossil records indicate that more-prolific reefs existed when seawater temperature were <2°C higher than today’s levels [9]. With seawater temperatures increasing at high latitudes, this coral community could possibly develop into a reef in the future. Moreover, past climatic data and climatic models suggest that seawater temperatures are increasing faster along the path of the KC [31], and that global warming may enhance heat transport by KC [15], further emphasizing this effect.

*Acropora* species dominated the coral community (Figure 1b). *Acropora* spp. tend to dominate Indo-Pacific reefs at low latitudes [6]. Their sensitivity to environmental disturbances has often led these species to be considered losers when confronted with environmental changes [44]. They are usually considered to be more tropical, which justifies their absence at higher latitudes such as at Iki (33.8°N) and Tsushima islands (34°N) [13], [34]. Two species, *A. hyacinthus* and *A. solitaryensis,* were particularly abundant in our survey (together comprising 48.36% of the benthic community), and contributed >80% to the coral cover (Table 1). *Acropora hyacinthus*, like other species such as *A. muricata*, is considered a key reef-forming species in tropical Indo-Pacific regions [45], [46]. However, with high mortality when the low temperature dips below 15°C during winter [9], [47], *A. hyacinthus* is also considered one of the most sensitive coral species to low temperatures along coastal Japan. In 1931–1932, *A. hyacinthus* was reported from the Ryukyus Archipelago and more-southerly locations, but not around Shikoku or more-northerly areas [48]–[50]. Although diving limitations could had been an obstacle for underwater surveys in the early 20^th^ century, the occurrence of the same species at different locations recorded by the same authors may be used as a good indicator of past distributions, particularly for shallow-water species. Today, *A.hyacinthus* represents almost 60% of the coral cover at the study site. This obvious species would likely have been recorded in the region if a similar coverage had existed back in the 1930s. However, if we consider the subgenus *Polystachys* Brook 1893 within the genus *Acropora* and the synonymy existing between *Madrepora sinensis* and *A. hyacinthus* [29], a specific morph of *A. hyacinthus* might have been reported as *A.* (*Polystachys) sinensis* in this region [48]–[50]. This suggests that *A. hyacinthus* may have existed in this region in low abundance in the 1930s. The first reliable record of *A. hyacinthus* in the Shikoku region is 1965 [51], [52], after which this species was regularly recorded during surveys [19], [53]–[55]. Therefore we may suspect relatively recent colonization and/or an increase in the abundance of *A. hyacinthus* in the Shikoku region some time between the 1930s and 1960s, which was reported by Yamano et al. [13] as poleward migration.

Compared to slowly growing species such as massive Faviidae or Poritidae corals [56], *A. hyacinthus* is a branching species that is subject to frequent fragmentation and usually needs a shorter time to recover from disturbances [57]. A recent study carried out at Sesoko Island in the Ryukyus Archipelago also highlighted the reproductive and growth capacities of *Acropora* species, notably *A. hyacinthus*, leading to those species being reconsidered long-term winners [58]. This may especially be applicable in high-latitude areas, which may represent particularly good refugia for sensitive and endangered tropical species with a high capacity for migration and colonization. Thirty-nine to 45 species of scleractinian corals (belonging to 23 genera) were reported from Misaki in 1931–1932, <2 km away from Tatsukushi [48]–[50]. Although diving limitations are likely influencing species diversity records in the 1930s and the taxonomic status of the species recorded needs to be revised, the present biodiversity at Tatsukushi (96 species for 40 genera [21]) and secure identifications may suggest the possible migration of some tropical taxa toward more northerly latitudes.

All functional groups (this term with others is defined in Table 2), except the ‘unattached’ functional group (free-living or solitary corals), were represented in the present coral community (Figure 4a). In term of species occurrences, the dominant functional group was composed of massive corals which is congruent with what is recorded on the Great Barrier Reef (dotted line in figure 4a; [28]). In Australia, massive corals comprise of about 31% of the species identified. At Tatsukushi, this group comprised up to 39% of the coral species, with >35% belonging to the family Faviidae. Faviidae species are known to have a wide latitudinal distribution [59] and the capacity to establish themselves beyond the limit of most other species [34], [35]. Diversity in coral life forms is usually used to describe potential functionalities offered by a coral community (e.g., [28]), but it is only when the abundance is considered in parallel can the interpretation of morphological diversity truly reflect the functionalities of a community. Considering species abundances/coverage (Figure 4b), however, clearly shows the major contribution of the ‘plate-like’ functional group instead, which is explained by the over-dominance of plate-like species such as *A. hyacinthus* and *A. solitaryensis*. In the current survey, the contrast between functionality explained by species diversity and by species coverage is a characteristic worth emphasizing, as it might indicate a type of coral community located in an expansion area of coral reef distribution or a community experiencing post-disturbance recovery. In both cases, opportunistic and pioneer species benefit from environmental changes. This might be considered a ‘pioneer frame-building phase’ [60], which produces system heterogeneity allowing the settlement of other coral species.

**Table 2:** Summary of definitions of terms used in this article.

Term	Definition
***Functional group***	Group of organisms contributing to the same particular functional process in an ecosystem.
***Functional redundancy***	Ability of an organism to carry out the same functional process as another under the same environmental conditions. The presence of multiple organisms within each functional group increases the functional redundancy and may have important implications for ecosystem stability. Redundant organism may be thus substitutable with little impact on ecosystem processes.
***Functional diversity***	Variety of functional processes in a particular ecosystem - *i.e.,* diversity in functional groups.
***Species diversity***	Variety and abundance of organisms inhabiting a given area.
***Resilience***	From an ecological point of view, the capacity of an ecosystem to recover its original functional diversity after being disturbed.

**Figure 5:**
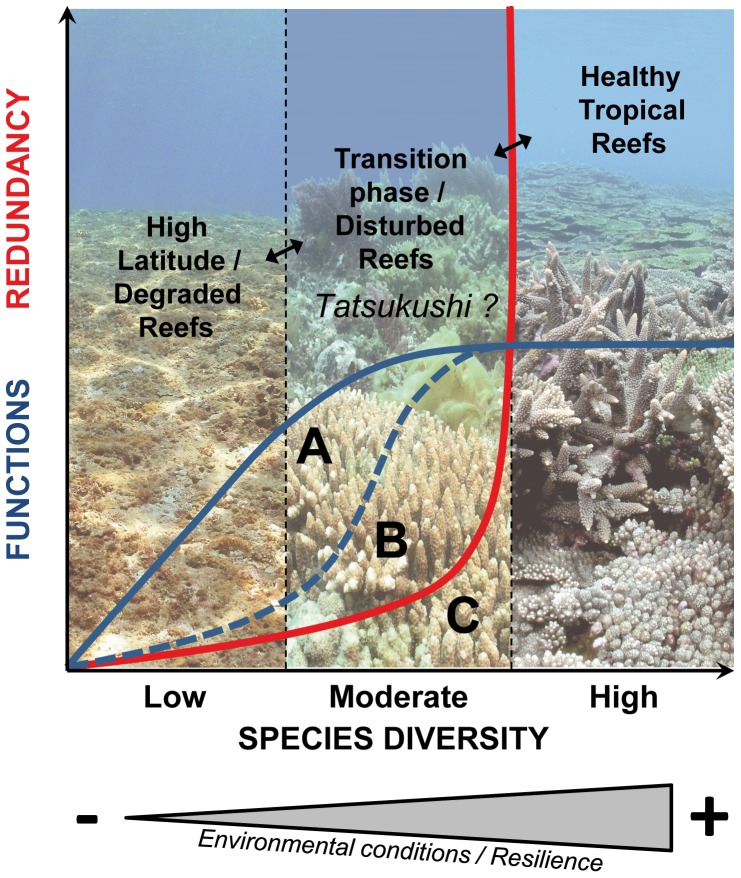
Relationship between the diversity and functional diversity (A and B)/redundancy (C): schematic scenarios (modified from [**61**] and [**62**]). A: Functional diversity increases at declining rates with increased species diversity, which reaches an asymptote at high diversity levels; B: high functional redundancy at low species diversity followed by a rapid increase at an intermediate species diversity, until functional diversity asymptotes at high diversity levels [49]. Photo credits: CM Hsu.

A conceptual view which depicts the potential relationship between functional diversity/redundancy and species diversity is proposed in Figure 5. Two scenarios were considered for functional diversity: (A) functional diversity showing a large increase at a low species diversity, then a slighter increase as the diversity becomes higher; and (B) a limited set of functions at low diversity (simple environment), then an abrupt increase in functions as the environment becomes more complex and diversified [61]. Both (A & B) might [61] or might not [62] reach an asymptote. Functional redundancy (C) was considered to increase at high levels of diversity, a pattern characteristic of highly diverse assemblages [62]. Species thus become strongly redundant only past a certain threshold. Theoretically, environmental conditions that maintain healthy and diversified tropical reefs offer a large number of functions where species are highly redundant. As environmental conditions become less optimal, species diversity decreases, leading to reductions in functional diversity and redundancy, eventually reducing the resilience capacity. Alternatively, this may also characterize a community in transition, as hypothesized here. Finally, low-diversity assemblages, where new species are likely to offer a new function to the system, may be characteristic of high-latitude communities or degraded reefs. A low resilience capacity makes these areas particularly sensitive to disturbances.

In high-latitude coral communities, lower temperatures and higher nutrient concentrations usually favor the growth of algae [8]. Tropical reefs usually sustain high primary productivity and turnover rates of turf algae [63]–[65], whereas in high-latitude areas, macroalgae prosper [66], [67]. Hence, the importance of herbivory is likely to be amplified, as it is known to have profound effects on the abundance and diversity of coral assemblages, by moderating competitive interactions between corals and macroalgae [68], [69].

A survey conducted in 2010 [25] found the fish community to largely be dominated by carnivorous fishes (73.6% of species and 93.7% of individuals). Maintaining herbivorous fish populations may have been limited by the low productivity of the algae assemblage (accounting for only 2% of the benthic cover) and/or alternatively by problems related to their digestive mechanisms in cooler waters. Abundances of some large herbivorous fishes (scarids and acanthurids) along the Brazilian coast present drastic declines at high latitudes due to temperature-dependent physiological constraints [70]. A possible explanation for the absence of macroalgae could be the high density of sea urchins (12.9±3.3 ind m^−2^), which seem to largely benefit from the prevailing environmental conditions. *Echinometra* spp. comprised the main urchin species in the coral-dominated area, while *Echinostrephus molaris* tends to dominate in areas devoid of live corals. Sea urchins are critical for controlling macroalgae [71]. They help maintain coral cover by limiting the competitiveness of macroalgae with corals. On the other hand, at high densities, they may threaten the coral community because of their bioeroding activity [72] and their grazing upon newly settled corals [73]. However, the net effect of a high urchin density to coral communities seems to be relatively positive in temperate waters because of the more-prolific growth of macroalgae in these areas [74]. In addition, Acroporid species may have further expanded through fragmentation of coral colonies due to physical impacts from typhoons because of their relatively rapid growth/recovery rates [57].

In 2001, corals at Tatsukushi suffered particularly high mortality after sedimentation following heavy rains [75]. In 2002, a restoration project at Tatskushi began by removing sediments from the bay that had accumulated after the runoff of 2001. Subsequent management actions consisted of stabilizing uphill soils through revegetation programs in the watershed. In parallel, an eradication program of natural predators such as *Drupella* spp. and *Acanthaster plancii* was launched by the Tatsukushi Marine Park to avoid possible outbreaks of these organisms. For example, 100,000∼200,000 individuals of *Drupella* were collected per year on the west coast of Shikoku at the peak of the extermination program from 1995∼2000 [75]. Both actions may have helped increase the coral cover these past few decades.

A benthic structure and composition such as those described herein are quite exceptional for a coral community located beyond the accepted geographical limits of coral reef development. Although this marginal community falls short of providing the functionalities and functional redundancy offered by healthy tropical reefs, its benthic structure can provide a base upon which a reef could eventually develop. Since some species seem to be benefiting from temperature increases, this community may still be undergoing a transition phase, with a strong potential to develop into a functional coral reef. Indeed, historical records show that it was a reef in the geological past [17], and fundamental modifications might already be occurring in regions at the periphery of coral reef distributions [2], [12], [13]. Rapid migration seems to be derived from diverse responses by individual species. While the existence of this scenario is clearly identified for terrestrial organisms [23], for corals it is still under debate and has to be stringently demonstrated [2]. As poleward-flowing currents may play an essential role in the dispersion and survival of coral species, it is essential to understand patterns of connectivity among populations along latitudinal gradients. However, a consequence of corals finding refuge at high latitudes might entail cascading effects on the resilience of other temperate ecosystems (e.g., kelp forests), which is important when considering the large-scale management of marine ecosystems.

## Supporting Information

Table S1 **Coral species recorded in September 2011 at the study site at Tatsukushi (Shikoku Island, Japan).**
(XLSX)
